# Three Novel Pigmentation Mutants Generated by Genome-Wide Random ENU Mutagenesis in the Mouse

**DOI:** 10.1002/cfg.382

**Published:** 2004-03

**Authors:** Vicky Tsipouri, John A. Curtin, Pat M. Nolan, Lucie Vizor, Claire A. Parsons, Colin M. Clapham, Ian D. Latham, Lesley J. Rooke, Jo E. Martin, Jo Peters, A. Jackie Hunter, Derek Rogers, Sohaila Rastan, Steve D.M. Brown, Elizabeth M.C. Fisher, Nigel K. Spurr, Ian C. Gray

**Affiliations:** 1 GlaxoSmithKline Pharmaceuticals New Frontiers Science Park Harlow CM19 5AW UK; 2 Department of Neurogenetics Imperial College London W2 1PG UK; 3 MRC Mammalian Genetics Unit and Mouse Genome Centre Harwell OX11 0RD UK; 4 Department of Histopthology Queen Mary and Westfield College London E1 4NS UK; 5 National Institutes of Health, Bethesda National Human Genome Research Institute (NHGRI) Bethesda MD 20892-8004 USA; 6 UCSF Cancer Center 2340 Sutter St. Room N461 Box 0808 San Francisco CA 94143-0808 USA; 7 63 Fordhook Avenue Ealing London W5 3LS UK; 8 Department of Neurodegenerative Disease National Hospital for Neurology & Neurosurgery Queen Square London WC1N 3BG UK; 9 Paradigm Therapeutics Ltd 214 Cambridge Science Park Cambridge CB4 0WA UK

## Abstract

Three mutant mice with pigmentation phenotypes were recovered from a genomewide random mouse chemical mutagenesis study. White toes (*Whto*; MGI:1861986), Belly spot and white toes (*Bswt*; MGI:2152776) and Dark footpads 2 (*Dfp2*; MGI:1861991) were identified following visual inspection of progeny from a male exposed to the point mutagen ethylnitrosourea (ENU). In order to rapidly localize the causative mutations, genome-wide linkage scans were performed on pooled DNA samples from backcross animals for each mutant line. *Whto* was mapped to proximal mouse chromosome (Mmu) 7 between *Cen* (the centromere) and *D7Mit112* (8.0 cM from the centromere), *Bswt* was mapped to centric Mmul between *D1Mit214* (32.1 cM) and *D1Mit480* (32.8 cM) and *Dfp2* was mapped to proximalMmu4 between *Cen* and *D4Mit18* (5.2 cM). *Whto*, *Bswt* and *Dfp2* may provide novel starting points in furthering the elucidation of genetic and biochemical pathways relevant to pigmentation and associated biological processes.


**Comparative and Functional Genomics**
*Comp Funct Genom* 2004; **5**: 123–127.
Published online in Wiley InterScience (www.interscience.wiley.com). **DOI:** 10.1002/cfg.382

**Short Communication**

### Three novel pigmentation mutants generated by genome-wide random ENU mutagenesis in the mouse

Vicky Tsipouri[1,2,a†*], John A. Curtin[1,2b†], Pat M. Nolan[3], Lucie Vizor[3], Claire A. Parsons[1], Colin M. Clapham[1], Ian D. Latham[1], Lesley J. Rooke[1], Jo E. Martin[4], Jo Peters[3], A. Jackie Hunter[1], Derek Rogers[1], Sohaila Rastan[1c], Steve D.M. Brown[3], Elizabeth M.C. Fisher[2d], Nigel K. Spurr[1] and Ian C. Gray[1e]

[1]*GlaxoSmithKline Pharmaceuticals, New Frontiers Science Park, Harlow CM19 5AW, UK*
[2]*Department of Neurogenetics, Imperial College, London W2 1PG, UK*
[3]*MRC Mammalian Genetics Unit and Mouse Genome Centre, Harwell OX11 0RD, UK*
[4]*Department of Histopathology, Queen Mary and Westfield College, London E1 4NS, UK*

**Correspondence to:*
*Vicky Tsipouri, National Institutes of Health, National Human Genome Research Institute (NHGRI), Bethesda, MD 20892-8004, USA.*
*E-mail: vtsip@mail.nih.gov*

[a]*Current address: National Institutes of Health, National Human Genome Research Institute (NHGRI), Bethesda, MD 20892-8004, USA.*

[b]*Current address: UCSF Cancer Center, 2340 Sutter St. Room N461, Box 0808, San Francisco, CA 94143-0808, USA.*

[c]*Current address: 63 Fordhook Avenue, Ealing, London W5 3LS, UK.*

[d]*Current address: Department of Neurodegenerative Disease, National Hospital for Neurology & Neurosurgery Queen Square, London WC1N 3BG, UK.*

[e]*Current address: Paradigm Therapeutics Ltd, 214 Cambridge Science Park, Cambridge CB4 0WA, UK.*

†*These authors contributed equally to this work.*

Received: 5 June 2003
Revised: 9 December 2003
Accepted: 19 December 2003

### Abstract

**Three mutant mice with pigmentation phenotypes were recovered from a genome-wide random mouse chemical mutagenesis study. White toes (*Whto*; MGI:1861986), Belly spot and white toes (*Bswt*; MGI:2152776) and Dark footpads 2 (*Dfp2*; MGI:1861991) were identified following visual inspection of progeny from a male exposed to the point mutagen ethylnitrosourea (ENU). In order to rapidly localize the causative mutations, genome-wide linkage scans were performed on pooled DNA samples from backcross animals for each mutant line. *Whto* was mapped to proximal mouse chromosome (Mmu) 7 between *Cen* (the centromere) and *D7Mit112* (8.0 cM from the centromere), *Bswt* was mapped to centric Mmu1 between *D1Mit214* (32.1 cM) and *D1Mit480* (32.8 cM) and *Dfp2* was mapped to proximal Mmu4 between *Cen* and *D4Mit18* (5.2 cM). *Whto, Bswt* and *Dfp2* may provide novel starting points in furthering the elucidation of genetic and biochemical pathways relevant to pigmentation and associated biological processes. Copyright © 2004 John Wiley & Sons, Ltd.**

**Keywords:** mouse; pigmentation mutant; ethylnitrosourea; mutagenesis; linkage

Copyright © 2004 John Wiley & Sons, Ltd.

### Introduction

Pigment arises from the melanosome, an organelle found in melanocytes that fills with melanin. The melanin is subsequently transferred from the melanocyte to surrounding keratinocytes, resulting in pigmentation (see Jackson, 1997). Defects in pigmentation can arise at the site of melanin production as a consequence of abnormal melanosomes, or as a result of developmental failure to deliver melanocytes at the required sites for correct pigmentation to occur. Melanocytes arise from the neural crest; during embryonic development, as the neural tube folds and closes, neural crest cells migrate from the dorsal neural tube to disparate regions of the embryo. Neural crest cells are precursors to a number of different cell types, including the neuronal and glial cells of the peripheral nervous system as well as skeletal and connective tissue components of the head, in addition to melanocytes (Nicholls, 1992). Consequently, mutations in genes affecting pigmentation can cause defects in other cell lineages if such cells share a common precursor with melanocytes.

To date, mouse genetic studies have implicated more than 40 proteins in the biochemical and developmental processes involved in pigmentation, including G-protein coupled receptors and their ligands, receptor tyrosine kinases and their ligands, melanogenic enzymes and transcription factors, to name a few (see Jackson, 1997; Nakamura *et al*., 2002). Although analysis of mutant mouse phenotypes has made an enormous contribution toward elucidating biochemical and developmental pathways involved in pigmentation, and more broadly in characterizing mammalian gene function in general, there is a clear disparity between the number of mouse phenotypes available and the number of known genes. If the entire spectrum of available mouse mutants for all observed phenotypes is considered, presently it is likely that mutant phenotypes exist for less than 10% of the predicted number of mouse genes (Brown and Hardisty, 2003). In order to fully exploit the mouse as a model organism for genetic research, there is a need to discover new phenotypes that result from mutations at new loci and uncover multiple mutants for the same locus. Several mutagenesis programmes have been established to increase the mutant mouse resource utilizing the mutagen ENU (Balling, 2001; Brown and Balling, 2001). ENU is a potent producer of point mutations; male mice exposed to ENU by intraperitoneal injection can acquire germline base changes at a level of $>5 \times 10^{-4}$/kb (Coghill *et al*., 2002). Because ENU mutagenesis acts in a random, genome-wide fashion, it is necessary to locate mutated genes by linkage analysis and positional cloning following recovery of mutant progeny from ENU-exposed males. The MRC/GSK mouse mutagenesis programme, initiated by the UK Medical Research Council and GlaxoSmithKline Pharmaceuticals (Nolan *et al*., 2000), has thus far generated over 160 dominant phenotypes by ENU mutagenesis and the chromosomal localization of over 70 of these phenotypes has been elucidated (**http://www.mgu.har.mrc.ac.uk/mutabase/**).

In this paper we describe three novel mutant mice with dominant coat colour phenotypes that were identified from the MRC/GSK ENU programme, White toes *(Whto)*, Belly spot and white toes *(Bswt)* and Dark footpads 2 *(Dfp2)*, and report their genetic map locations as determined by backcross linkage analysis.

### Materials and methods

### Animals

Animal studies described here were carried out under the guidance issued by the Medical Research Council in *Responsibility in the Use of Animals for Medical Research* (July 1993) and Home Office Project Licence No. 30/1517. Details of the mutagenesis programme are described elsewhere (Nolan *et al*., 2000). Briefly, BALB/c males (Charles River, UK) were injected intraperitoneally with two weekly doses of 100 mg/kg ENU (Sigma) at approximately 10 weeks of age. Visual examination was carried out on $F_1$ progeny of mutagenized male mice crossed to C3H/HeH females (Charles River, UK) and at 5 weeks of age, the mice were assessed using the SHIRPA behavioural and functional assessment protocol (Rogers *et al*., 1997). For inheritance testing, $F_1$ mice were backcrossed to the C3H/HeH strain and progeny classified for the phenotype identified in the founder. Mutant lines were subsequently maintained by backcrossing to C3H/HeH.

### Linkage Analysis

Preliminary linkage analysis was performed on pooled DNA samples, as described elsewhere

Copyright © 2004 John Wiley & Sons, Ltd. 

(Isaacs *et al.*, 2000). Briefly, tail snips were taken from mutant backcross mice and DNA extracted using standard procedures. DNA concentrations were measured by UV absorbance and equimolar aliquots of DNA combined for each strain. Non-mutant backcross pools were also constructed for *Dfp2*; for *Bswt* and *Whto* mutant pools only were used. The DNA pools, together with DNA from BALB/c, C3H/HeH and $F_1$ mice, were screened with 93 simple tandem repeat markers polymorphic between BALB/c and C3H/HeH, spaced at 20 cM (centimorgan) or less, selected from the Whitehead/MIT database (**www-genome.wi.mit.edu**). Genotyping was performed using an Applied Biosystems 377 PRISM system in accordance with the manufacturer's instructions. Genotype data were analysed using a modified version of TrueAllele software (Cybergenetics) and linkage assessed by relative BALB/c;C3H/HeH allele signal strength. Preliminary linkage was confirmed by genotyping individual animals. Crude map locations were refined by genotyping with additional markers.

### Results

Three pigmentation mutants were identified in the progeny of BALB/c male mice exposed to ENU and crossed to C3H/HeH females; autosomal dominant inheritance of each phenotype was confirmed by backcross analysis. The resulting mutant lines were named 'White toes', 'Belly spot and white toes' and 'Dark footpads 2' (Table 1). White toes (*Whto*, MGI:1 861 986) is characterized by a white belly spot and white hind toes, with hydrocephaly in some mice and small litter size. Belly spot and white toes (*Bswt*, MGI:2 152 776) has white feet and a white belly spot. Dark footpads 2 (*Dfp2*, MGI:1 861 991) is similar to the previously known dark footpads mutant (*Dfp*; Kelly, 1968) and, as the name suggests, has markedly increased pigmentation in the footpads. At 5 weeks of age, *Bswt* and *Dfp2* mice were assessed using the SHIRPA behavioural and functional assessment protocol (Rogers *et al.*, 1997). This screen, involving a battery of 40 simple tests, is semi-quantitative and based on modifications of earlier screens described by Irwin (1968). No behavioural or functional anomalies were detected in either mutant line. The results of the SHIRPA tests and a full description of the protocol can be viewed at **http://www.mgu.har.mrc.ac.uk/mutabase/**

In order to rapidly identify regions of linkage, genome-wide scans using 93 simple tandem repeat markers with spacing of 20 cM or less, selected from the Whitehead/MIT database (**http://www-genome.wi.mit.edu/**: marker details available on request), were performed on pools of equimolar DNA samples from mutant (BALB/c × C3H/HeH) × C3H/HeH backcross animals for *Whto* ($n = 44$) and *Bswt* ($n = 81$), as described in Isaacs *et al.* (2000). Both mutant and non-mutant DNA pools were screened for *Dfp2* ($n = 31$ and 20, respectively). Mutant pools only were used for *Whto* and *Bswt* because, unlike *Dfp2*, these lines showed evidence for incomplete penetrance in the ratio of mutant vs. non-mutant backcross animals recovered. From these initial genome scans, *Bswt* was mapped to centric (*Mus musculus*; Mmu) chromosome 1, *Dfp2* was mapped to proximal Mmu 4 and *Whto* was mapped to proximal Mmu 7. These crude map positions for *Whto* and *Dfp2* were originally reported in Nolan *et al.* (2000); *Bswt* has not been reported previously.

The map positions were confirmed by genotyping individual mice and more precise locations identified by genotyping with additional markers in the critical interval for each mutant. Map positions were refined to *D1Mit214* (32.1 cM)–*D1Mit480* (32.8 cM) for *Bswt, Cen* (0.0 cM)–*D4Mit18* (5.2 cM) for *Dfp2* and *Cen* (0.0 cM)–*D7Mit112* (8.0

**Table 1.** Phenotypic descriptions and map positions for *Bswt, Dfp2* and *Whto*

| Name | Symbol | Phenotype | Map position[1] |
|---|---|---|---|
| Belly spot and white toes | *Bswt* | Belly spot and white feet, occasional tail band | *D1Mit214* (32.1 cM)–*D1Mit480* (32.8 cM) |
| Dark footpads 2 | *Dfp2* | Dark footpads | *Cen* (0.0 cM)–*D4Mit18* (5.2 cM) |
| White toes | *Whto* | White hind toes, belly spot, occasional hydrocephaly, small litter size | *Cen* (0.0 cM)–*D7Mit112* (8.0 cM) |

[1] Marker positions based on the Jackson genetic map (**http://www.informatics.jax.org**).

Copyright © 2004 John Wiley & Sons, Ltd. 

cM) for *Whto* (Table 1). Marker positions are taken from the Jackson mouse genetic map (**http://www.informatics.jax.org**).

### Discussion

No previously mapped pigmentation mutants listed in the mouse genome database (**www.informatics.jax.org**) lie in the genetic intervals bearing *Whto* or *Bswt*, other than the reduced pigmentation mutant *rp*, which maps within the *Whto* region (Gibb *et al*., 1981). The *rp* mutant shows generalized reduction in pigmentation and is recessive, whereas the dominant *Whto* has a distinct white belly spot and white hind toes; therefore it is unlikely that *Whto* and *rp* are allelic. The gene responsible for the *rp* phenotype has yet to be identified. Although *Whto* and *Bswt* appear to be unique, Fitch *et al.* (2003) recently described *Dsk4*, a pigmentation mutant strikingly similar to *Dfp2*. *Dsk4* maps to an interval on proximal chromosome 4 that is coincident with *Dfp2*, strongly suggesting that *Dfp2* and *Dsk4* are allelic. Combining data from *Dfp2* and *Dsk4* should therefore expedite mapping and gene identification for this mutant. The map location of the original *Dfp* mutant (Kelly, 1968) is unknown; therefore it is possible that *Dfp* and *Dfp2/Dsk4* are allelic. However, Fitch *et al.* (2003) also describe several other dark footpad mutants and it is equally likely that *Dfp* is an allele at one of these alternative loci.

Although no comparable pigmentation mutants map to the same genetic regions as *Whto* or *Bswt*, mutants similar to these, either mapping to other regions or as yet unmapped, have been described previously. The splotch (*Sp*) mutant (Russell, 1947) shares many of the characteristics of the *Whto* and *Bswt* phenotypes. Mice that are heterozygous for *Sp* have a white patch on their abdomen; mouse embryos homozygous for *Sp* show defects in neural tube closure and neural crest cell development, leading to reduction or absence of a number of neural crest cell derivatives and resulting in death at embryonic day 16 (Franz, 1989; Serbedzija and McMahon, 1997). The *Sp* phenotype is caused by mutations in the *Pax3* gene, located on Mmul (Epstein, 1991). Although *Bswt* is also linked to Mmul, *Pax3* at 44 cM from the centromere according to the Jackson map (**www.informatics.jax.org**), lies outside the critical interval (*Bswt* is between 32.1 and 32.8 cM on the Jackson map). *Bswt* does not therefore appear to be allelic to *Sp*. *Bst* (belly spot and tail; Southard and Eicher, 1977) is a second dominant mutant with similarities to *Bswt* and *Whto*. Heterozygotes have ventral spotting, a short kinked tail and occasionally white feet, reduced body size, malocclusion, anomalies of the spine and eyes, and polydactyly. *Bst* maps to Mmul6 (Rice *et al*., 1995) and therefore is not allelic with either *Bswt* or *Whto*.

Significant deviation from the expected ratio of 1 mutant:1 non-mutant for a fully penetrant dominant trait was observed for *Bswt* and *Whto* (81 : 115 and 44 : 65, respectively) giving an estimated penetrance of approximately 80% for both lines at the first backcross generation. This reduced penetrance may result from inter-animal variation in genetic background as a consequence of heterogeneity with respect to the BALB/c and C3H/HeH contribution to the genome of each mouse; certain genetic background combinations may mask the effect of the mutant allele. However, it is also possible that increased mortality of mutant mice *in utero* may be the cause of the skew in favour of non-mutant animals. An alternative explanation for an apparent reduction in penetrance is that two or more ENU-generated point mutations are required for expression of the phenotype and that segregation of these mutations occurs in the backcross; however, in this instance such an explanation is unlikely, as the expected outcome would be an perceived drop in penetrance to 50% or less.

Given the phenotypic similarities between *Bst, Bswt, Sp* and *Whto*, it is possible that they operate on the same biochemical or developmental pathway. As described in the introduction, pigmented cells originate in the neural crest; much is known about the migratory pathways taken by neural crest cells and their derivatives, but our knowledge of the genes that control these processes, although increasing rapidly, is still limited (Maschhoff *et al*., 2000; Wu *et al*., 2003). Given that *Bswt* and *Whto* are phenotypically similar to *Sp* and show a heterogeneous distribution of pigmentation (white spotting), it is likely that these mutants result from defects of melanocyte development or migration, rather than melanosome dysfunction, and may be useful in the elucidation of genetic and biochemical pathways relevant to

Copyright © 2004 John Wiley & Sons, Ltd. 

developmental processes involving the neural crest. As yet, *Bswt* and *Whto* homozygote mutants have not been generated; this is an important next step and may reveal developmental defects and provide further clues to *Bswt* and *Whto* gene function.

Although the genetic intervals harbouring *Whto* and *Dfp2*, at approximately 8.0 cM and 5.2 cM, respectively, are too large to speculate usefully on potential candidate genes in these regions; the *Bswt* region at less than 1 cM is more tractable. 19 genes are listed in the interval bounded by the *Bswt* flanking markers *D1Mit214* and *D1Mit480* in the February 2002 freeze of the University of California Santa Cruz (UCSC) mouse genome sequence browser (**http://genome.ucsc.edu/**). Approximately 40 genes are listed in the equivalent human genome interval (November 2002 freeze), which has more complete coverage. Of these, the most intriguing candidate for the *Bswt* phenotype is the Frizzled-7 gene (*Fzd7*). Frizzled proteins are cell surface receptors for Wnt ligands; activation of the Wnt–Frizzled signalling pathway ultimately results in Wnt–dependent gene expression. Experiments in *Xenopus* have established a role for Wnt–Frizzled signalling in neural crest formation, and melanocyte induction by Frizzled-3 has been demonstrated (see Wu *et al.*, 2003). Complete ablation of Fzd7 in *Xenopus* results in severe gastrulation defects (Winklbauer *et al.*, 2001), but the effects of partial or complete loss of Fzd7 function in mammalian systems is unknown. We are currently screening *Fzd7* for point mutations in *Bswt* mice.

### References

Balling R. 2001. ENU mutagenesis: analysing gene function in mice. *Annu Rev Genom Hum Genet* **2**: 463–492.

Brown SDM. 1998. Mouse models of genetic disease — new approaches, new paradigms. *J Inherit Metab Dis* **21**: 532–539.

Brown SD, Balling R. 2001. Systematic approaches to mouse mutagenesis. *Curr Opin Genet Dev* **11**: 268–273.

Brown SD, Hardisty RE. 2003. Mutagenesis strategies for identifying novel loci associated with disease phenotypes. *Semin Cell Dev Biol* **14**: 19–24.

Epstein DJ, Vekemans M, Gros P. 1991. *Splotch (Sp2H)*, a mutation affecting development of the mouse neural tube, shows a delection within the paired homeodomain of Pax-3. *Cell* **67**: 767–774.

Fitch KR, McGowan KA, van Raamsdonk CD, *et al.* 2003. Genetics of dark skin in mice. *Genes Dev.* **17**: 214–228.

Franz T. 1989. Persistent truncus arteriosus in the Splotch mutant mouse. *Anat Embryol* **180**: 457–464.

Gibb S, Hakansson EM, Lundin LG, Shire JG. 1981. Reduced pigmentation (rp), a new coat colour gene with effects on kidney lysosomal glycosidases in the mouse. *Genet Res* **37**: 95–103.

Isaacs AM, Davies KE, Hunter AJ, *et al.* 2000. Identification of two new Pmp22 mouse mutants using large-scale mutagenesis and a novel rapid mapping strategy. *Hum Mol Genet* **9**: 1865–1871.

Kelly EM. 1968. Dark foot pads, *Dfp. Mouse News Lett* **38**: 31.

Maschhoff KL, Baldwin HS. 2000. Molecular determinants of neural crest migration. *Am J Med Genet* **97**: 280–288.

Nakamura M, Tobin DJ, Richards-Smith B, Sundberg JP, Paus R. 2002. Mutant laboratory mice with abnormalities in pigmentation: annotated tables. *J Dermatol Sci* **28**: 1–33.

Nicholls JG, Martin AR, Wallace BG. 1992. *From neuron to brain: a cellular and molecular approach to the function of the nervous system*, 3rd edn. Sinauer Associates: Sunderland, MA; 342–344.

Nolan PM, Peters J, Strivens M, *et al.* 2000. A systematic, genome-wide, phenotype-driven mutagenesis programme for gene function studies in the mouse. *Nature Genet* **25**: 440–443.

Rice DS, Williams RW, Ward-Bailey P, *et al.* 1995. Mapping the Bst mutation on mouse chromosome 16: a model for human optic atrophy. *Mamm Genome* **6**: 546–548.

Russell WL. 1947. *Splotch*, a new mutation in the house mouse, *Mus musculus*. *Genetics* **32**: 102.

Serbedzija GN, McMahon AP. 1997. Analysis of neural crest cell migration in *splotch* mice using a neural crest-specific *lacZ* reporter. *Dev Biol* **185**: 139–147.

Southard JL, Eicher EM. 1977. *Bst. Mouse News Lett* **56**: 40.

Winklbauer R, Medina A, Swain RK, Steinbeisser H. 2001. Frizzled-7 signalling controls tissue separation during *Xenopus* gastrulation. *Nature* **413**: 856–860.

Wu J, Saint-Jeannet JP, Klein PS. 2003. Wnt-frizzled signaling in neural crest formation. *Trends Neurosci* **26**: 40–45.

Copyright © 2004 John Wiley & Sons, Ltd. 
